# The first imported case of melioidosis in a patient in central China

**DOI:** 10.1080/22221751.2019.1654839

**Published:** 2019-08-20

**Authors:** Youhua Yuan, Zonghui Yao, Erhui Xiao, Jiangfeng Zhang, Baoya Wang, Bing Ma, Yi Li, Wenjuan Yan, Shanmei Wang, Qiong Ma, Junhong Xu, Yuming Wang, Enguo Fan

**Affiliations:** a Department of Clinical Microbiology, Henan Provincial People's Hospital, People’s Hospital of Zhengzhou University, People’s Hospital of Henan University, Zhengzhou, People’s Republic of China; b Department of Infectious Disease, Henan Provincial People's Hospital, People’s Hospital of Zhengzhou University, People’s Hospital of Henan University Zhengzhou, People’s Republic of China

**Keywords:** Melioidosis, *Burkholderia pseudomallei*, whole-genome sequence, multilocus sequence type, emerging infectious disease

## Abstract

Here, we report the first imported case of melioidosis from Laos in central China. COMPACT VITEK2 identification system and PCR, as well as sequencing methods confirmed that the patient was infected by *Burkholderia pseudomallei*, a bacterial species closely related to an isolate detected in Thailand. These findings are highly valuable for an early diagnosis, treatment and to prevent the spread of this emerging infectious disease in central China.

Melioidosis is a serious, fatal, and infectious disease caused by the Gram-negative bacteria *Burkholderia pseudomallei* [1]. Its clinical manifestations range from skin abscess to overwhelming sepsis and death. Melioidosis is highly endemic to Southeast Asia, and northern Australia [2,3]. However, since it has not been reported in central China, its diagnosis is extremely challenging for local clinicians. The prevalence of melioidosis in China is unknown, although new endemic foci have been identified in Hainan province, southern China, and sporadic cases have been reported in China [4]. Here, we report a case of melioidosis in a traveller who returned to central China from Laos.

On 9 October 2017, a 54-year-old Chinese man with poorly controlled type 2 diabetes sought medical care at Henan Provincial People’s Hospital. He had been living in Laos as a tunnel worker for ten years and visited the hospital seeking treatment for intermittent fever and abdominal pain, which lasted for approximately two months. At Laos, he was diagnosed as dengue and treated for two months at a local hospital. He also underwent treatment at other hospitals in Henan province but with poor therapeutic results. His body temperature fluctuated between 38.3°C and 38.9°C, mainly in the afternoon. Upon admission to our hospital’s infection department for managing his irregular fever and left upper abdominal pain, preliminary examination indicated that the highest temperature was 38.9°C. His abdomen was soft with tenderness in the upper-left portion and knocking pain around the spleen area. Additionally, laboratory tests showed that the patient had signs of a bacterial infection and uncontrolled diabetes (Figure 1(A)). Abdominal computed tomography (CT) revealed an enlarged spleen with multiple low-density shadows suggesting a spleen abscess. These symptoms, in combination with the case that the patient had a history of diabetes and was from a melioidosis epidemic area, strongly hint a probability of melioidosis. To examine this suspicion, 8 bottles of blood samples were consecutively collected from different sites of the patient with an interval of 1 day. While the first test was negative, samples from two bottles showed positive in the second test for *B. pseudomallei*. Therefore, the antimicrobial imipenem was administered. However, the pain in the left upper abdomen increased further and another CT scan of the abdomen was performed, which showed that the patient had splenic vein emboli and a possible infarct; the spleen was irregular in shape and less dense (Figure 1(B)). The interventional and vascular surgery departments recommended to perform a continuing anti-infection treatment and conduct an exploratory laparotomy when necessary. After 25 days of treatment with intravenous imipenem, fever, abdominal pain, and other uncomfortable symptoms disappeared. The patient became stabilized with a negative blood test results. The patient was also requested to orally take doxycycline and sulfonamides for another 6 months. Subsequent regular follow-up showed that the patient remained healthy for 1 year after completing the melioidosis therapy. This study was approved by the Research Ethics Committee of Henan Provincial People’s Hospital. Informed consent of this patient was not required. Figure 1.(A) Laboratory test results of the patient. (B) Abdomen computed tomography shows splenic vein emboli and a possible infarct; the spleen is seen to be irregular in shape and low in density. (C) A gram-stained direct smear of blood culture showed negative short bacilli with intense staining at both ends and light staining in the middle (magnification: 1000×). (D) Gel electrophoresis of multi-locus sequence type, specific, and 16S rRNA identification for *B. pseudomallei* isolated from the patient. M:DNA Marker; 1:lipA; 2:nark; 3:gltB; 4:acE; 5:lepA; 6:ndH; 7:gmhD; 8:Tat; 9:16SrRNA. (E) Chromatogram of 16SrRNA sequencing for *B. pseudomallei* isolated from the patient. (F) Chromatogram of specific gene Tat sequencing for *B. pseudomallei* isolated from the patient. (G) Alignment result of 16SrRNA sequence from NCBI BLAST database for *B. pseudomallei* isolated from the patient. (H) Alignment result of specific gene Tat sequence from NCBI BLAST database for *B. pseudomallei* isolated from the patient. (I) Whole-genome phylogeny of sequence type 46 *B. pseudomallei* isolates obtained from the patient (**arrow**). Analyses of isolates from other countries are also shown.

**Figure:**
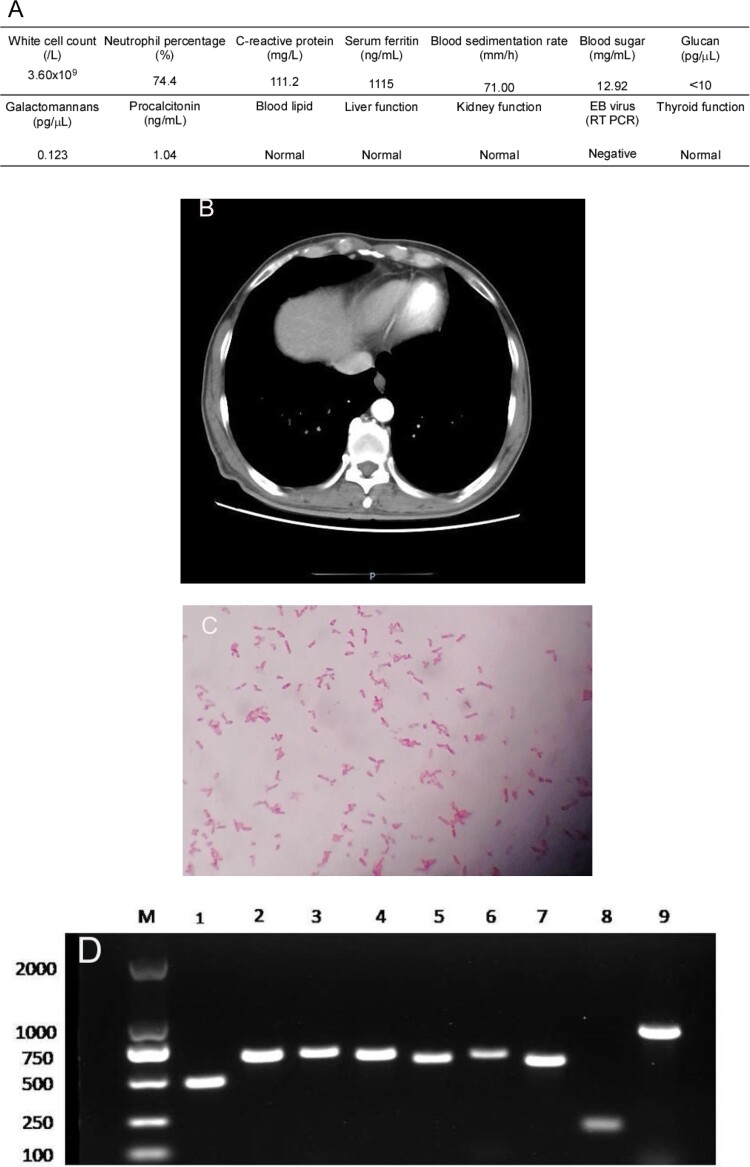

**Figure:**
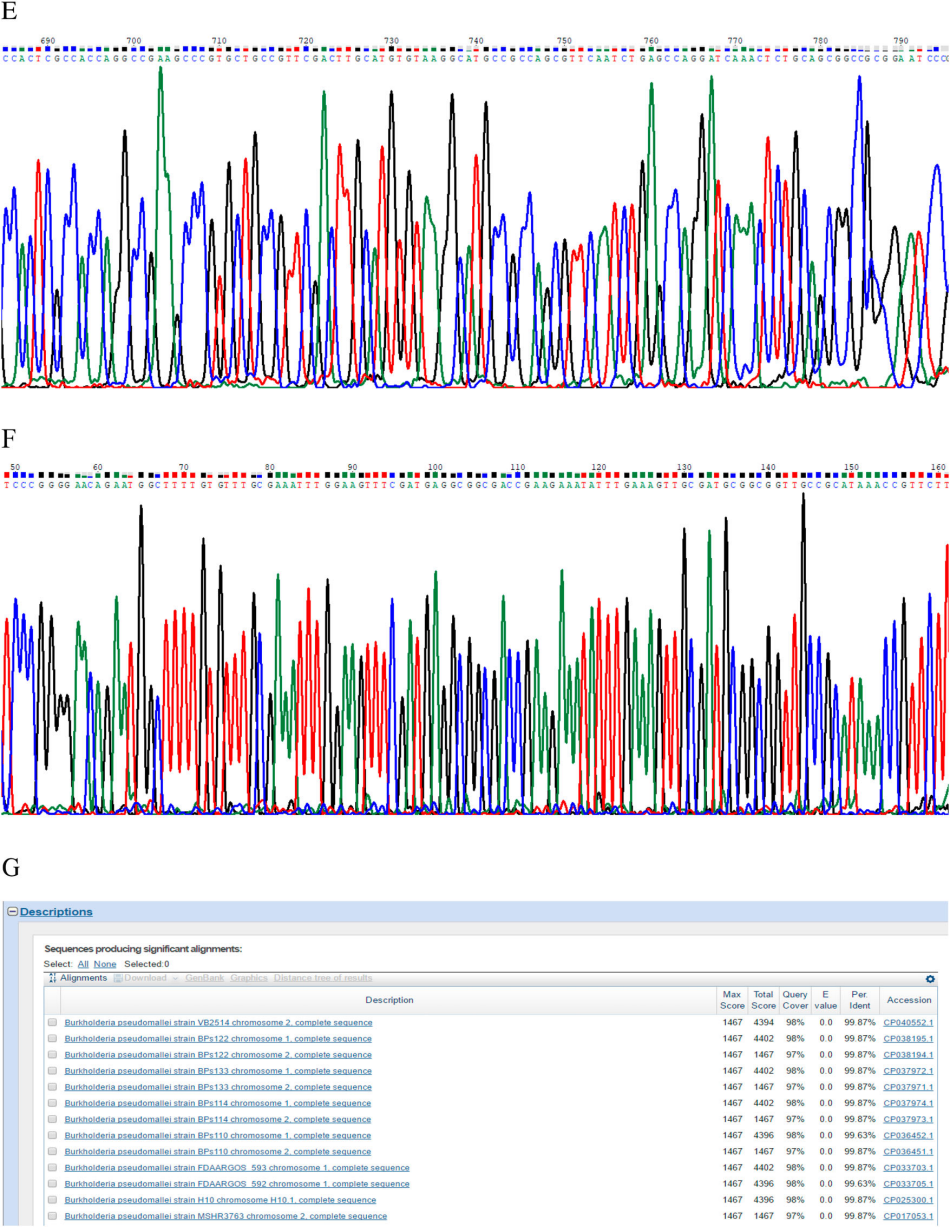

**Figure:**
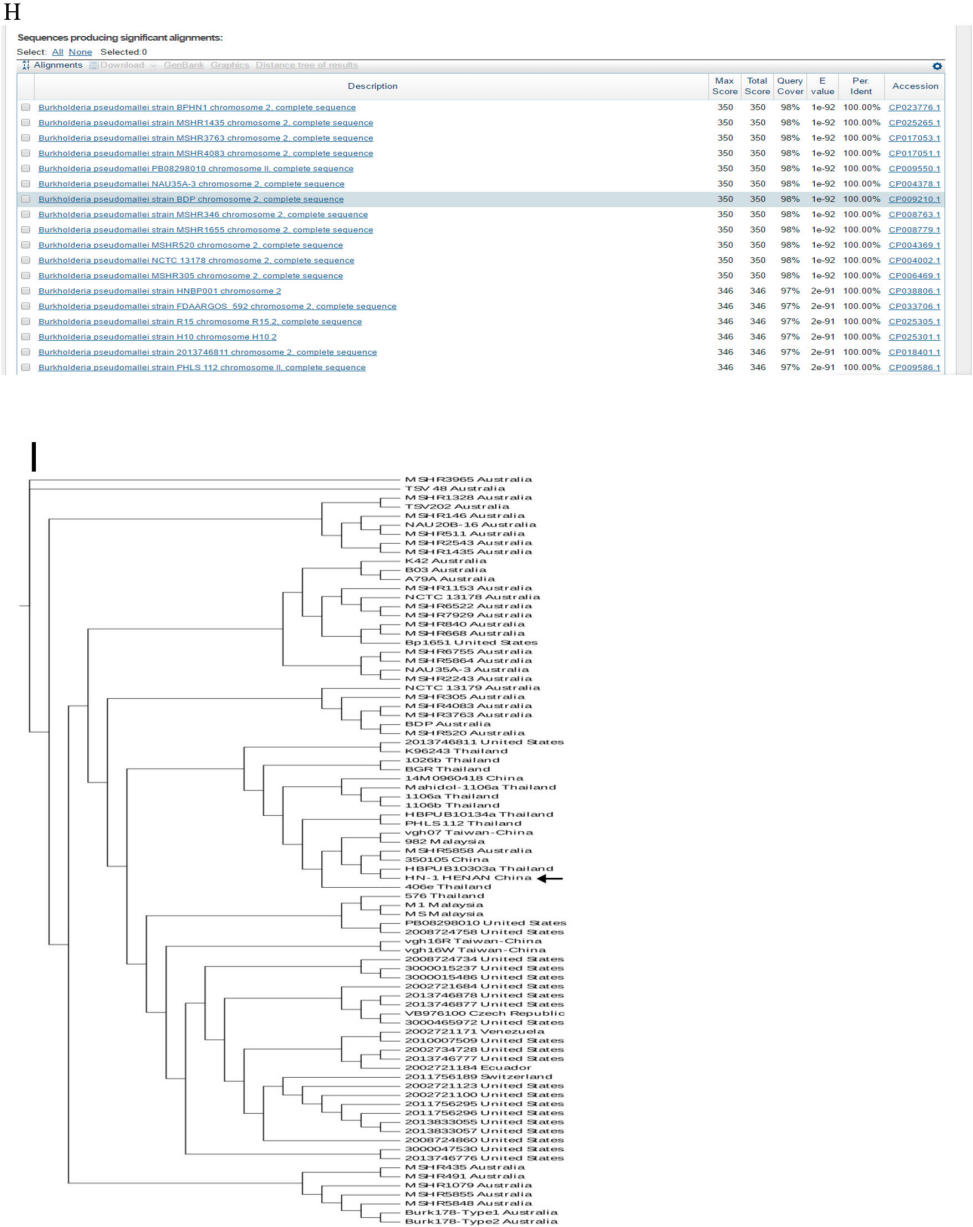

The two bottles of admitted blood cultures grew Gram-negative rods after 30 h of incubation. Short bacilli with an intense staining at both ends and a light staining in the middle were visualized in the gram-stained direct smear blood culture (Figure 1(C)). The isolate grew on blood, MacConkey, and chocolate agar when subcultured based on Gram staining, the isolate was found to be the same kind of bacteria as in the original culture. The strain was later identified as *B. pseudomallei* by conventional phenotype methods, using COMPACT VITEK2 identification system (BioMerieux Ltd., France). Furthermore, the PCR results using specific primers (LPW13372: 5′-CAA GAA CGGTTT ATG CG-3′ and LPW13373: 5′-GAA GTG ATC CAT CAA ATGTC-3′) and 16S rRNA amplification (Fd1:5′CCGAACGTCGACAACAGAGTTTGATCCTGGCTCAG-3′and rp2: 5′-CCCGGGATCCAAGCTT ACGGCTACCTTGTTACGACTT-3′) for sequence analysis confirmed that the isolate was *B. pseudomallei* [5,6] (Figure 1(D–H)). Whole-genome sequencing conducted by Shanghai Novel Bioinformatics Company, China, further verified the isolate as *B. pseudomallei*. The drug susceptibility test showed that the isolate was susceptible to imipenem, amoxicillin/clavulanic acid, trimethoprim/sulfamethoxazole, and ceftazidime, but resistant to aminoglycosides and cephalosporins.

To type the identified isloate, multilocus sequence typing (*MLST*) was conducted and it was turned out that this isolate can be grouped as sequence type 46 (ST46) (https://pubmlst.org/) (Figure 1(D)), which is consistent with those isolates that originated from Laos [7]. To further define the relatedness of ST46 isolates, whole-genome sequencing was performed. To this end, genomic DNA was extracted using the LAIFENG DNeasy cell and bacteria kit (LAIFENG, Shanghai, China) according to the manufacturer’s instructions. The sample was sequenced at Novel Bioinformatics Co., Ltd (Shanghai, China) using HiSeq 2000 (Illumina, San Diego, CA, USA). Denovo assembly was performed with SPANDx version 3.10 using the *B. Pseudomallei* strain K96243 from the NCBI database (http://bpseudomal­lei.mlst.net/) as the reference genome. The current known whole-genome sequences of all other *B. Pseudomallei* strains were downloaded from the National Centre for Biotechnology Information (NCBI) website (https://www.ncbi.nlm.nih.gov/genome/) and were used to construct a phylogenetic tree. Whole-genome identification of single-nucleotide polymorphisms (SNPs) was performed by MUMmer Version 3.9.4 alpha, followed by a phylogenetic construction using RAxML v8.2.9 with the GTR+ nucleotide substitution model, respectively [8]. The results confirmed that the isolate was most closely related to that of ST48 from Thailand [9] (Figure 1(I)).

Clinical diagnosis of melioidosis in non-endemic areas is extremely challenging because the signs of this disease are non-specific and similar to those of other common diseases, such as pneumonia and dengue. Laboratory diagnosis is also challenging. In the present case, *B. pseudomallei* grew readily in culture. However, the Phoenix 100 Identification System (BD Company, USA) or matrix-assisted lasers desorption/ionization time of flight mass spectrometry (Bruker Co. Ltd., Germany) could not identify the species definitively. To make these methods potentially useful for identifying *B. pseudomallei*, the database needs to be optimized by adding reference spectra for this organism and close relatives (e.g. *B. thailandensis*). *B. pseudomallei* differ from other *Burkholderia* spp. [10] in its pathogenicity and epidemiology. Unfortunately, it can be easily misidentified as *B. Thailandensis* and *B. cepacia* complex based on phenotypic tests [11].

Epidemiological data from different countries support the hypothesis that inhalation and injection is the most possible predominant transmission route of *B. Pseudomallei* under severe weather events (e.g. storms and typhoons) [12]. A study from Taiwan reported an air sampling technique that uses a filtration real-time quantitative PCR method to quantify ambient *B. pseudomallei* DNA; high positive rates were found during typhoons [12]. Presumably, the subject of our study was infected through air or soil in Laos, where *B. pseudomallei* is endemic [13]*.* To date, no other case of melioidosis has been reported from Henan province.

In summary, *B. pseudomallei* infection should be suspected in patients with chronic fever who have travelled to endemic areas, particularly to Southeast Asian tropical countries [14]. The significance of this case report is that doctors in non-endemic areas may easily misdiagnose melioidosis as tuberculosis, pneumonia or sepsis, which thus significantly delay the treatment or even lead to a death. Specific symptoms of this disease are multiple abscesses, especially in the lungs, liver and spleen together with a long-term fever. Therefore, if a patient is from endemic areas and have a long-term fever together with single or multiple sites of abscesses in the body combined with immunodeficiency baseline disease, the first suspicion should be this disease, which demands a confirmation by several times of blood culture test. A key treatment is to against the pathogen according to the drug sensitivity test and general treatment guidelines by use of correct antibiotics. A subsequent consolidation treatment must last for at least a half year until a complete absorption of the abscess before ending the treatment in order to avoid an incomplete treatment, which will result in a relapse of this disease. Doctors in non-endemic areas must be very sensitive of this disease because the mis-diagnosis derived mortality rate is 40–60% [15]. Moreover, because of the risk of transmission to laboratory workers and potentials using *B. pseudomallei* for bioterrorism, clinical laboratories should perform only limited tests on the suspected isolates before referring them to a professional biosafety laboratory for more definitive verification.
